# Guanine nucleoside alleviates mycophenolic acid-induced toxicity in mouse embryonic stem cells

**DOI:** 10.3389/fcimb.2025.1689743

**Published:** 2025-12-03

**Authors:** Baoshan Lin, Ying Ta, Dandan Ou, Xiaolong Liu, Xiaoqiang He, Zhun Rang, Dongqiang Zhang, Wei Fu, Daoliang Lan

**Affiliations:** 1 College of Animal Husbandry and Veterinary Medicine, Southwest University for Nationalities, Chengdu, China; 2 Animal Disease Prevention and Control Center of Aba Tibetan and Qiang Autonomous Prefecture, Markang, China; 3 Science Technology and Agriculture and Animal Husbandry Bureau of Hongyuan County, Hongyuan, China; 4 Longri Stud Farm, Hongyuan, China

**Keywords:** MPA, mouse embryonic stem cells, GUO, proliferation, apoptosis, differentiation, rescue

## Abstract

**Introduction:**

Mycophenolic acid (MPA), an immunosuppressant widely used in organ transplantation and the treatment of autoimmune diseases, poses a teratogenic risk to mammalian fetuses. Guanine nucleoside (GUO), an important intermediate in purine metabolism, has the potential to modulate immune responses.

**Methods:**

In this study, we systematically evaluated the effects of MPA at various concentrations (0.05, 0.075, 0.1 μM) on the proliferation, apoptosis,and differentiation of mouse embryonic stem cells (PGK12.1), and investigated whether GUO at different concentrations (2, 4, 8 mM) could mitigate MPA induced toxicity.

**Results:**

The results showed that MPA inhibited the viability of PGK12.1 cells in a dose dependent manner, increased reactive oxygen species (ROS) levels, reduced mitochondrial membrane potential, significantly down regulated the expression of proliferation-related genes (PCNA, CCND1, CDK1) and the antiapoptotic gene Bcl2, up regulated apoptotic genes (Bax, Caspase3), and disrupted the differentiation potential of PGK12.1 cells. Notably, treatment with 8 mM GUO significantly ameliorated these toxic effects. RNA-seq analysis revealed that pathways associated with cell proliferation, apoptosis, and differentiation (including TGF-b, PI3K-AKT, and p53) were significantly enriched in the MPA-treated group, suggesting that these signaling pathways may be involved in the response to the MPA-induced phenotype, whereas GUO may potentially counteract these effects through the regulation of pathways associated with chromatin architecture and cytoskeletal organization.

**Discussion and conclusion:**

This study elucidates the toxic effects of MPA on mouse embryonic stem cells and highlights the protective role of GUO, providing a foundation for further investigations into the mechanisms underlying MPA induced toxicity.

## 1 Introduction

Stem cells have emerged as a major focus in life sciences, offering vast potential for both basic cell biology and clinical applications. Embryonic stem cells (ESCs), derived from early embryos or primordial germ cells, possess self-renewal and pluripotent differentiation potential, making them an ideal model for studying fetal development before and after implantation (Wu and Izpisua, 2016). Mouse embryonic stem cells (mESCs), derived from the inner cell mass of blastocysts and cultured *in vitro*, retain the ability to differentiate into all three germ layers: endoderm, mesoderm, and ectoderm (Evans and Kaufman, 1981). The *in vitro* self-renewal and differentiation capabilities of mESCs provide a unique advantage for developmental toxicity testing (Rezvanfar et al., 2016). In 2000, the European Centre for the Validation of Alternative Methods (ECVAM) validated the Embryonic Stem Cell Test (EST) as an alternative method for evaluating chemical-induced embryotoxicity (Spielmann et al., 1997). In addition, stem cell-based differentiation models can rapidly detect early changes induced by environmental contaminants and exhibit high sensitivity and accuracy in developmental toxicity and teratogenicity assays (Zhang et al., 2025). Therefore, investigating the toxic effects and underlying molecular mechanisms of Penicillium derived metabolites during embryonic development using the mESC model is of great importance.

MPA, a key secondary metabolite isolated from Penicillium mycophenolicum, exhibits antiinflammatory, antimicrobial, and anticancer activities (Bentley, 2000). MPA is widely used as an immunosuppressant for treating autoimmune diseases and preventing immune rejection following organ transplantation (Girman et al., 2020). Previous studies have demonstrated that MPA inhibits inosine monophosphate dehydrogenase (IMPDH), thereby disrupting *de novo* guanine synthesis and suppressing lymphocyte proliferation (Cicinnati et al., 2009). However, the U.S. Food and Drug Administration (FDA) initially classified mycophenolate mofetil (MMF) as a Category C pregnancy drug, indicating that while adverse fetal effects were observed, potential risks could not be excluded due to insufficient human data (Pisoni and D’Cruz, 2008). Animal studies have demonstrated that oral administration of MMF at 1 mg/kg/day caused anophthalmia, brain herniation, and umbilical herniation in rat offspring, while administration at 80 mg/kg/day in rabbits, without maternal toxicity, led to fetal malformations (Anderka et al., 2009; Eckardt and Stahlmann, 2010). Notably, autoimmune diseases are more prevalent in women, and administration of MMF to women of childbearing age may increase the risk of miscarriage and fetal malformations (Anderka et al., 2009).

Nucleoside metabolism plays a crucial role in alleviating immunodeficiency and regulating autoimmune responses (Abt et al., 2022). GUO, a key nucleoside metabolite, which can bypass the MPA-induced blockade in purine synthesis. In higher eukaryotes, inosine monophosphate dehydrogenase (IMPDH) is the rate-limiting enzyme in *de novo* GTP synthesis and a potential target for antiviral, anticancer, antimicrobial, and immunosuppressive therapies (Braun-Sand and Peetz, 2010). Addition of guanosine monophosphate (GMP), the primary active product downstream of IMPDH, has been shown to reverse inhibition of cell proliferation (Fotie, 2018). Furthermore, guanine enhances plant pathogen resistance by inducing reactive oxygen species bursts, hemicellulose deposition, and MAPK phosphorylation (Wang et al., 2022). Current research on GUO has primarily focused on neurological disorders, with limited studies addressing their role in mitigating embryonic developmental toxicity.

In this study, we used mouse embryonic stem cells PGK12.1 as an *in vitro* model to investigate early embryonic development, with the aim of elucidating the toxicity phenotypes of MPA on embryonic stem cells, including proliferation inhibition, cell death, and differentiation disorders, and evaluating the role and possible mechanism of GUO as a candidate protective factor, providing an experimental basis for the further study of the toxicity mechanism of MPA.

## 2 Materials and methods

### 2.1 Cell culture and passaging

PGK12.1 is a mouse embryonic stem cell line derived from blastocysts and kindly provided by Prof. Neil Brockdorff’s laboratory. The cells were morphologically normal and free of contamination. They were cultured in Dulbecco’s Modified Eagle Medium/Nutrient Mixture F-12 (DMEM/F12) supplemented with 10% fetal bovine serum, 1% penicillin-streptomycin, 1% non-essential amino acids, 1% glutamine, 0.1 mM 2-mercaptoethanol, and 1000 U/mL leukemia inhibitory factor (LIF), and maintained in a humidified incubator at 37°C with 5% CO_2_. Cells were passaged when they reached 70–80% confluency and displayed healthy morphology.

### 2.2 MPA treatment and GUO rescue experimental design

The experiments were conducted in control, MPA-treated, and GUO-rescue groups. In the control group, 1% DMSO was added without MPA. The MPA-treated groups received final concentrations of 0.05 μM, 0.075 μM, or 0.1 μM MPA, with three replicates per concentration, ensuring that cell number and condition were consistent across all groups prior to treatment. The GUO rescue experiments included the control group (no MPA and GUO, 1% DMSO as vehicle), the MPA group (0.075 μM MPA without GUO), and three rescue groups: rescue A (0.075 μM MPA + 2 mM GUO), rescue B (0.075 μM MPA + 4 mM GUO), and rescue C (0.075 μM MPA + 8 mM GUO).

### 2.3 Cell viability assay

Cell viability was assessed using the Cell Counting Kit-8 (CCK-8; #AR1160, Boster). PGK12.1 embryonic stem cells were seeded in 96-well plates and incubated at 37°C with 5% CO_2_ and high humidity for 24, 48, and 72 hours. Ten microliters of CCK-8 solution were added to each well, gently mixed, and incubated for 1 hour. Absorbance was measured at 450 nm using a microplate reader, and OD values were analyzed after excluding the highest and lowest readings from each group. Each experiment was performed in triplicate, with six technical replicates per concentration.

### 2.4 RT-qPCR analysis

The housekeeping gene Gapdh was used as an internal control. Proliferation- and apoptosis-related mRNA sequences were retrieved from GenBank. Primers were designed using Primer Premier 5 software, and their specificity was verified using Primer-BLAST. All primers were synthesized by Sangyo Bioengineering Co. All primer sequences are listed in **Table 1**.

**Table 1:** 

Genes	Sequence (5’-3’)
*PCNA*	F: TTGCACGTATATGCCGAGACC; R: GGTGAACAGGCTCATTCATCTCT
*CCND1*	F: ACCCTGACACCAATCTCCT; R: CTCCTTCTGCACGCACTT
*CDK1*	F: GAACACCTTTCCCAAGTGGA; R: CCATTTTGCCAGAGATTCGT
*Bax*	F: TGAAGACAGGGGCCTTTTTG; R: AATTCGCCGGAGACACTCG
*Bcl2*	F: GTCGCTACCGTCGTGACTTC; R: CAGACATGCACCTACCCAGC
*Caspase3*	F: ACAGCACCTGGTTACTATTC; R: CAGTTCTTTCGTGAGCAT
*Gapdh*	F: TGCCCCCATGTTTGTGATG; R: TGTGGTCATGAGCCCTTCC
*Nestin*	F: GGCATCCCTGAATTACCCAA; R: AGCTCATGGGCATCTGTCAA
*Gata6*	F: CTTCTCCTTCTACACAAGCGACCA; R: ATACTTGAGGTCACTGTTCTCGGG
*Map2*	F: TTCTTTTGCTTGCTCGGGATT; R: ATACAGGGCTTGGTTTATTTCAGAGA
*Eomes*	F: CCCTATGGCTCAAATTCCAC; R: CCAGAACCACTTCCACGAAA
*Sox17*	F: ACTTGCTCCCCACAATCACT; R: ACCCCGCTGTTTGTGTTTAG
*Oct4*	F: CCCCAATGCCGTGAAGTTG; R: TCAGCAGCTTGGCAAACTGTT

### 2.5 Western blotting analysis

Adherent cells were washed with PBS, scraped from the culture dish using a cell scraper, and collected by centrifugation. Cells were lysed in RIPA buffer (#P0013E, Beyotime) on ice for 30 minutes. Protein concentration was determined using the bicinchoninic acid (BCA) assay (#P0012S, Beyotime). Protein samples were denatured by adding 5× loading buffer and heating at 95°C for 10 minutes. Proteins were separated by SDS polyacrylamide gel electrophoresis (SDS-PAGE), transferred to a PVDF membrane, blocked with 5% skimmed milk for 1 hour, and incubated with primary antibodies overnight at 4°C. Primary antibodies included anti-Bax (1:1000, #A00183, Beyotime), antiBcl-2 (1:1000, #M00181-2, Beyotime), anti-Caspase-3 (1:1000, #PB9188, Beyotime), and anti-PCNA (1:1000, #CPA9711, Cohesion Biosciences, UK). After washing with PBS containing Tween-20, membranes were incubated with HRP-conjugated secondary antibody (1:20, 000, #A0208, Bio-Rad) for 1 hour. Protein bands were visualized using an enhanced chemiluminescence (ECL) kit (#1705060, Bio-Rad) and imaged with a chemiluminescence detection system.

### 2.6 ROS detection

Intracellular reactive oxygen species (ROS) levels were measured using a ROS Detection Kit (#R6033, Shanghai BestBio, China). The fluorescent probe DCFH-DA was diluted 1:1000 in DMEM/F12 medium to a final concentration of 10 μM. PGK12.1 ESCs were cultured for 24 hours, washed with PBS, and incubated with the diluted DCFH-DA at 37°C in the dark under 5% CO_2_ and high humidity for 30 minutes. After washing the cells 2–3 times with serum-free DMEM/F12, fluorescence was observed under an inverted fluorescence microscope.

### 2.7 JC-1 mitochondrial membrane potential detection

The detection of JC-1 mitochondrial membrane potential was performed using a kit (#J6004S, Shanghai BestBio, China). An appropriate amount of JC-1 (100×) staining solution was diluted with ultrapure water, vortexed vigorously to ensure complete dissolution and mixing, and then mixed with Assay Buffer (10×) to prepare the JC-1 staining working solution. PGK12.1 ESCs were cultured in a 24-well plate for 24 hours to allow adherence. Afterward, 300 µL of JC-1 staining working solution was added to each well, with CCCP solution serving as a positive control. The cells were then incubated in a 5% CO2, 37°C humidified incubator for 15 minutes in the dark. Following incubation, the cells were washed twice with PBS buffer, and observed under an inverted fluorescence microscope for red and green fluorescence, with photographs taken accordingly. Normal mitochondria with intact membrane potential emit red fluorescence, while apoptotic or necrotic cells, where the dye exists in its monomeric form, emit green fluorescence.

### 2.8 RNA-Seq experiments

RNA-seq analysis was conducted on PGK12.1 ESCs treated with 0.075 μM MPA and 8 mM GUO, with three replicates per group. DESeq2 software was used to screen for differentially expressed genes between samples, and significantly differentially expressed genes were filtered based on thresholds of |log2FC| ≥ 1 and fdr < 0.05. The differential gene expression plot was generated using the ggplot2 package. Functional enrichment analysis and pathway enrichment analysis were performed using the cluster Profiler R package, based on the Gene Ontology (GO) database (http://www.geneontology.org) and the Kyoto Encyclopedia of Genes and Genomes (KEGG) database (http://www.genome.jp/kegg/). Bubble plots and bar charts were used to visualize the top 20 enriched GO terms and significant KEGG pathways (*P* < 0.05).

### 2.9 Statistical analysis

All experimental data were processed and statistically analyzed using GraphPad Prism version 9.0. Quantitative data are presented as mean ± standard deviation (SD). Statistical significance was defined as *P* < 0.05 or *P* < 0.01. Comparisons between two groups were conducted using a two-tailed Student’s t-test, while comparisons among multiple groups were assessed using one-way ANOVA. *Post hoc* pairwise comparisons were performed using the LSD t-test. All images were analyzed using ImageJ software, with fluorescence intensity, brightness, and cell area automatically quantified by the program.

## 3 Results

### 3.1 Effects of MPA on proliferation and apoptosis of PGK12.1 cell lines

**Figure 1:**
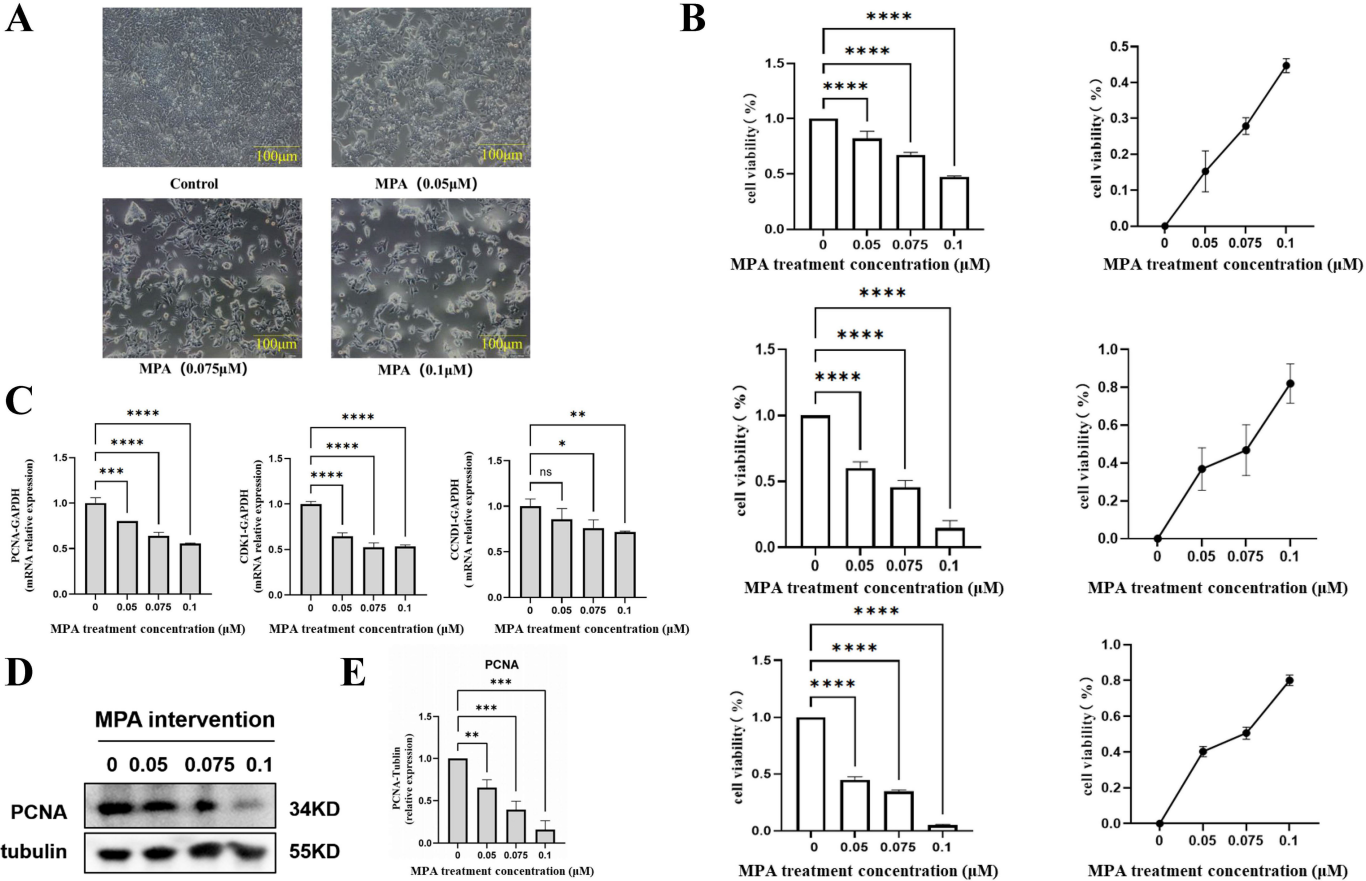
Effect of MPA on the proliferation of PGK12.1 cell line. **(A)** Morphological changes of PGK12.1 cell line after 24 h of MPA treatment (scale bar, 100μm); **(B)** Effects of different concentrations of MPA on cell viability and proliferation inhibition rate of PGK12.1 cells after 24 h, 48 h, and 72 h of treatment. **(C)** Detection of mRNA expression levels of cell proliferation related genes PCNA, CDK1, and CCND1 by RT-QPCR. **(D)** Detection of cell proliferation related protein PCNA expression by Western Blot; **(E)** Quantitative analysis of grayscale values of protein bands. * Indicates a statistically significant difference compared to the corresponding control group, with * representing *P* < 0.05, ** representing *P* < 0.01, *** representing *P* < 0.001, **** representing *P* < 0.0001, and ns indicating no difference and no statistical significance.

After gradient addition of MPA to the PGK12.1 cell line and culturing for 24 hours, we observed through cellular morphological changes that, with the increase in MPA dosage, especially in the 0.075 μM and 0.1 μM groups, the cells exhibited rupture, shrinkage, and vacuolization, with apoptotic bodies and cell debris floating in the culture medium (**Figure 1A**). Cell viability assays revealed that as the MPA concentration in-creased, cell viability gradually decreased, and cell proliferation was significantly inhibited (**Figure 1B**).

The mRNA expression of relevant genes and the corresponding protein expression in the treated cells were further examined using RT-QPCR and Western blotting methods after 24 hours. The results indicated that, compared with the control group, the mRNA expression levels of the proliferation genes PCNA, CDK1, and CCND1 were significantly decreased in the MPA treated experimental group. Furthermore, as the dose of MPA increased, the mRNA expression levels of these three genes showed a dose-dependent decrease, with statistical significance (*P* < 0.05, *P* < 0.01) (**Figure 1C**). Meanwhile, the relative expression of PCNA protein was down-regulated in a dose-dependent manner, with the most significant down-regulation observed in the experimental group treated with 0.1 μM MPA (**Figures 1D, E**).

**Figure 2:**
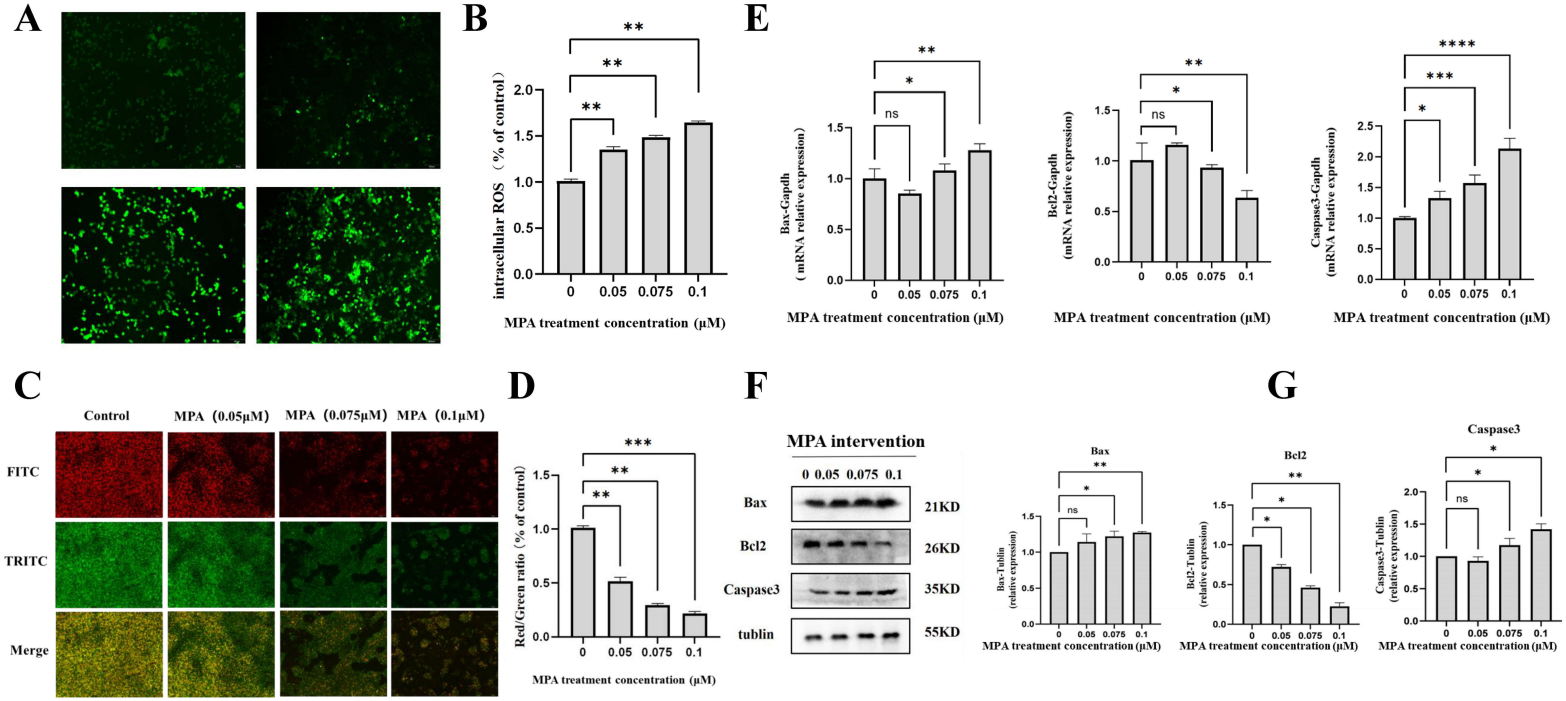
Effect of MPA on apoptosis of PGK12.1 cell line. **(A)** Fluorescent staining results of intracellular ROS content (scale bar, 100μm). **(B)** Analysis of ROS fluorescence intensity. **(C)** Fluorescent staining results of PGK12.1 ESC mitochondrial membrane potential using JC-1 (scale bar, 100μm). **(D)** Analysis of the red/green fluorescence intensity ratio of mitochondrial membrane potential. **(E)** mRNA expression levels of apoptosis-related genes Bcl2, Bax, and Caspase3 detected by RT-QPCR. **(F)** Detection of apoptosis-related protein expression by Western Blot. **(G)** Quantitative analysis of protein band grayscale values. * indicates a statistically significant difference compared to the corresponding control group, with * representing *P* < 0.05, ** representing *P* < 0.01, *** representing *P* < 0.001, **** representing *P* < 0.0001, and ns indicating no difference and no statistical significance.

Excessive ROS can induce cellular oxidative stress, and a decrease in mitochondrial membrane potential is a hallmark of early cell apoptosis. To investigate the effect of MPA on the apoptosis of PGK12.1 ESCs, we conducted assessments of ROS levels and mitochondrial membrane potential (ΔΨm). It was found that with the increase in MPA concentration, ROS levels significantly rose (**Figures 2A, B**). The green fluorescence signal of JC-1 monomers decreased in a dose-dependent manner with increasing MPA concentration, indicating a reduction in membrane potential. This decrease in membrane potential exacerbated mitochondrial damage and promoted cell apoptosis (**Figures 2C, D**).

The mRNA expression levels of apoptosis-related genes Bcl2, Bax, and Caspase3 were detected by RT-QPCR. It was found that the mRNA expression of the anti-apoptotic gene Bcl2 was significantly down regulated, while the mRNA expressions of the apoptotic genes were significantly up regulated. Moreover, the impact on the mRNA expression levels of these three genes increased sequentially with the increase in MPA (MPA) dosage, and the difference was statistically significant (*P* < 0.05). These results indicate that MPA can promote the expression of apoptotic genes in the PGK12.1 cell line (**Figure 2E**). Western blot results showed that the relative expression levels of the apoptotic proteins Bax and Caspase3 increased after MPA treatment, and this increase was dose-dependent with the increase in MPA concentration. The relative expression level of the anti-apoptotic protein Bcl2 decreased, also showing a dose-dependent decrease with the increase in MPA concentration (**Figures 2F, G**).

### 3.2 Effect of MPA on differentiation of PGK12.1 cell line

**Figure 3:**
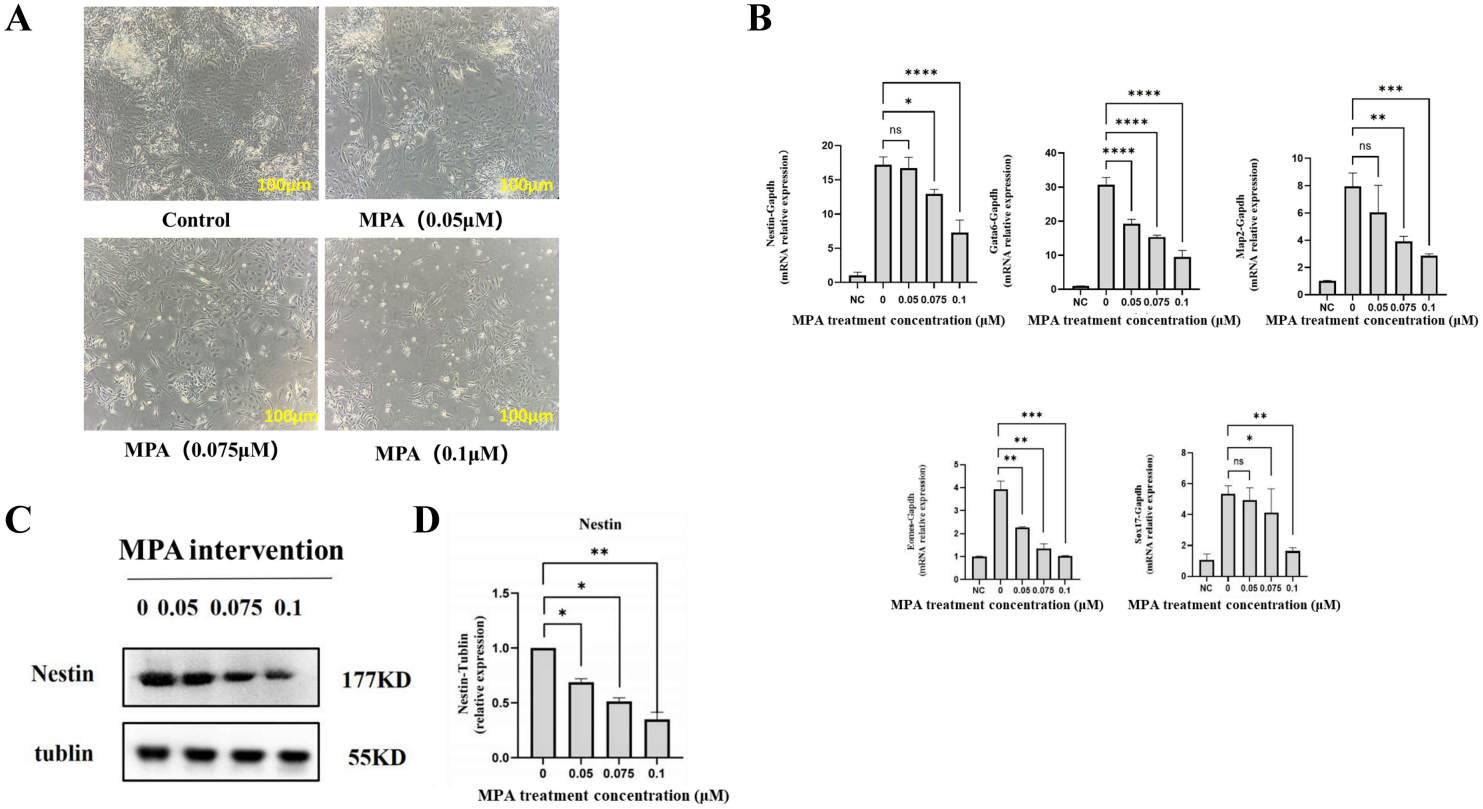
Effect of MPA on differentiation of PGK12.1 cell line. **(A)** Morphology of PGK12.1 cell line after 5 days of RA induction and differentiation (scale bar, 100μm). **(B)** RT-QPCR detection of mRNA expression levels of differentiation-related genes (ectoderm markers Nestin, Map2, endoderm markers Gata6, Sox17, and mesoderm marker Eomes). **(C)** Western Blot detection of changes in protein expression of the ectoderm marker Nestin. **(D)** Quantitative analysis of grayscale values of protein bands. * indicates a statistically significant difference compared to the corresponding control group, with * representing *P* < 0.05, ** representing *P* < 0.01, *** representing *P* < 0.001, **** representing *P* < 0.0001, and ns indicating no difference and no statistical significance.

Embryonic stem cells (ESCs) originate from the inner cell mass of the blastocyst and possess the ability to self-renew indefinitely and differentiate into all cell types of the three embryonic germ layers. To investigate the effect of MPA on the differentiation function of PGK12.1 ESCs, cells were treated with different concentrations of MPA (0.05, 0.075, and 0.1 μM) for 24 hours, followed by induction of differentiation using EMSD medium supplemented with 100 nM RA. The RA induction lasted for 3 days, during which small amounts of differentiated morphologies, characterized by “rosette”-like clusters, emerged in all groups. By day 4 of differentiation, differentiated cells were observed in each group. On day 5 of differentiation, a large number of clustered differentiated cells were observed in all groups, and the number of differentiated cells decreased sequentially with increasing MPA dosage (**Figure 3A**).

Meanwhile, cells treated with MPA for 48 hours and differentiated for 5 days were collected to detect the mRNA and related protein expression levels of marker genes for each germ layer. RT-QPCR results showed that compared with undifferentiated cells (NC group), the expression of the ectoderm markers Nestin and Map2, the endoderm markers Gata6 and Sox17, and the mesoderm marker Eomes were significantly increased in all differentiated cells, while their expression levels were sequentially reduced after MPA treatment (*P* < 0.05) (**Figure 3B**). Western blot results indicated that the relative expression level of the ectoderm marker Nestin protein was significantly decreased in MPA treated cells and showed a dose-dependent decrease with increasing concentrations of MPA (**Figures 3C, D**).

### 3.3 Rescue effect of GUO on MPA treated PGK12.1 cell line

Research has shown that under the influence of IMPDH inhibitors, the inhibition of cell proliferation can be reversed by the addition of GUO, GMP, GTP, or deoxy-GMP, with exogenous addition of GUO being the rescue method adopted by most researchers. In this study, PGK12.1 ESCs were simultaneously treated with gradient concentrations of GUO and MPA (0.075 μM) for 24 hours. CCK8 cell viability assay results showed that when the concentration of GUO was 0.1 mM, the viability of MPA treated PGK12.1 cell line increased slightly, but the statistical difference was not significant. At concentrations of 2, 4, and 8 mM, GUO significantly rescued PGK12.1 cells from apoptosis (*P* < 0.05), with a highly significant increase in cell viability at 8 mM GUO (*P* < 0.01).

**Figure 4.1:**
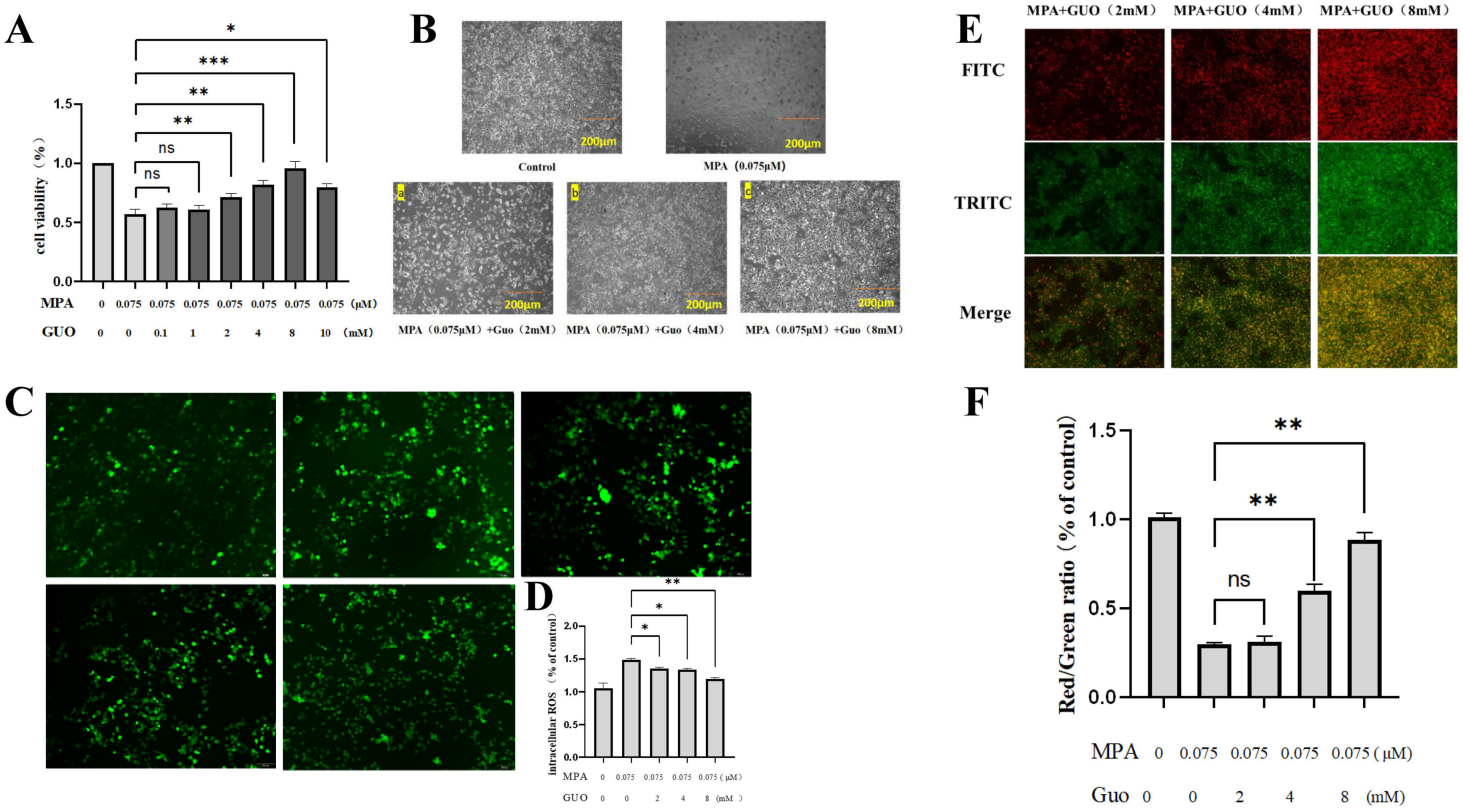
The rescue effect of GUO on MPA treated PGK12.1 cell line. **(A)** Cell viability detection after treating PGK12.1 with different concentrations of GUO + MPA (0.075 uM) for 24 hours. **(B)** The effect of GUO on MPA induced morphological changes in PGK12.1 ESCs (scale bar, 200μm). **(C)** Fluorescent staining results of intracellular ROS content (scale bar, 100μm). **(D)** Analysis of ROS fluorescence intensity. **(E)** JC-1 detection of mitochondrial membrane potential fluorescent staining results in PGK12.1 ESCs (scale bar, 100μm). **(F)** Analysis of the red/green fluorescence intensity ratio of mitochondrial membrane potential. * representing *P* < 0.05, ** representing *P* < 0.01, *** representing *P* < 0.001.

Therefore, these three concentrations were selected for subsequent experiments (**Figure 4.1A**). Observation of cell morphology revealed that GUO could effectively repair the morphological damage induced by MPA in mouse PGK12.1 ESCs (**Figure 4.1B**).

The results of ROS detection showed that the ROS content in the cells of the MPA treated group was significantly reduced (*P*<0.05), indicating a significant alleviating effect (**Figures 4.1C, D**). The results of the JC-1 method detection demonstrated that as the concentration of GUO increased, the Red/Green ratio of mitochondrial membrane potential in the PGK12.1 cell line continued to rise, suggesting that GUO had a significant improving effect on the decrease in mitochondrial membrane potential of the PGK12.1 cell line exposed to MPA. Among them, the mitochondrial membrane potential of cells treated with 8 mM GUO showed the highest recovery (**Figures 4.1E, F**).

**Figure 4.2:**
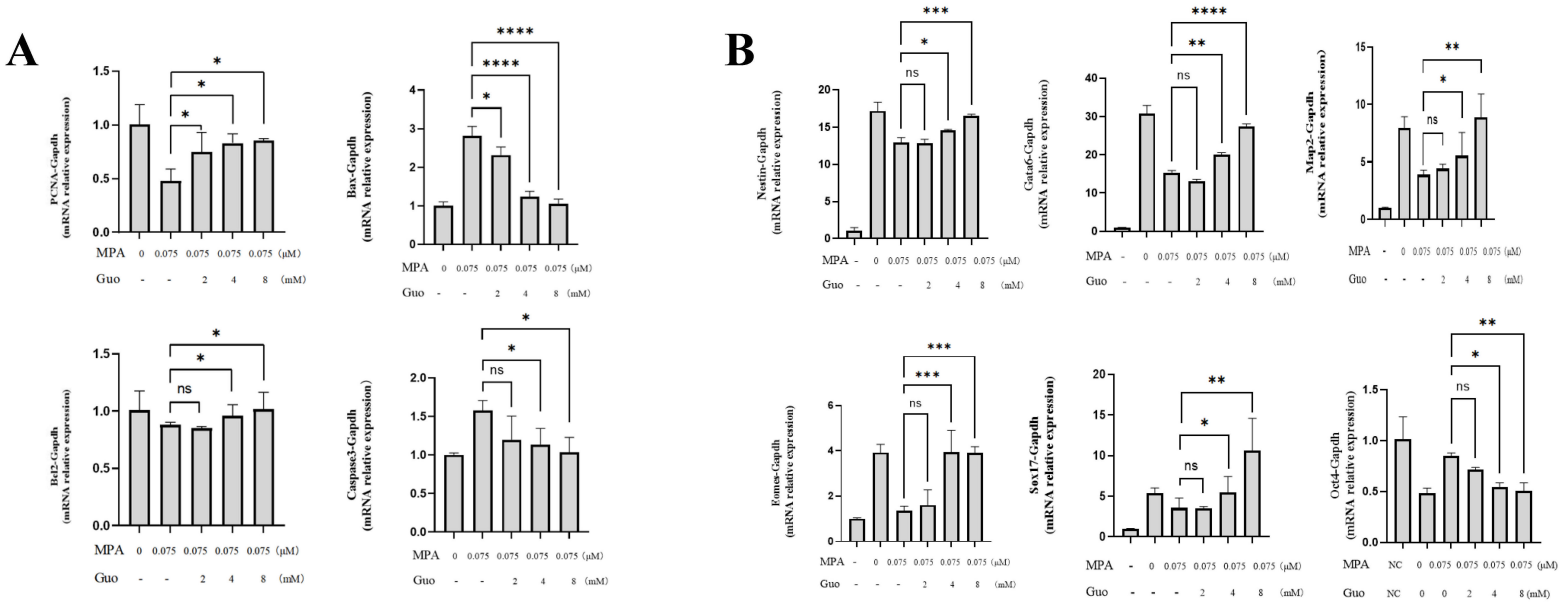
Detection of apoptosis and differentiation related gene expression after GUO’s intervention. **(A)** RTQPCR analysis of mRNA transcription levels of apoptosis-related genes PCNA, Bax, Bcl2, and Caspase3. **(B)** RTQPCR analysis of mRNA transcription levels of differentiation-related genes Nestin, Gata6, Eomes, Map2, and Sox17. ns, not significant; *P < 0.05, **P < 0.01, ***P < 0.001, ****P < 0.0001.

The results of detecting changes in mRNA expression levels of apoptosis-related genes PCNA, Bax, Bcl2, and Caspase3 using the RT-QPCR method showed that GUO’s treatment significantly down regulated the mRNA expression of Bax and Caspase3 (*P*<0.01) and significantly up regulated the mRNA expression of Bcl-2 and PCNA (*P*<0.05), indicating that GUO can alleviate MPA induced PGK12.1 ESC apoptosis (**Figure 4.2A**). The RT-QPCR results for detecting mRNA expression levels of the three germ layer marker genes Nestin, Gata6, Eomes, Map2, and Sox17 (**Figure 4.2B**) showed that GUO can significantly up regulate the expression of the three germ layer marker genes and down regulate the expression of the pluripotency marker Oct4 mRNA (*P*<0.05, *P*<0.01).

### 3.4 Elucidating the potential molecular mechanism of GUO’s improvement on MPA induced apoptosis in PGK12.1 ESC based on transcriptome analysis

**Figure 5:**
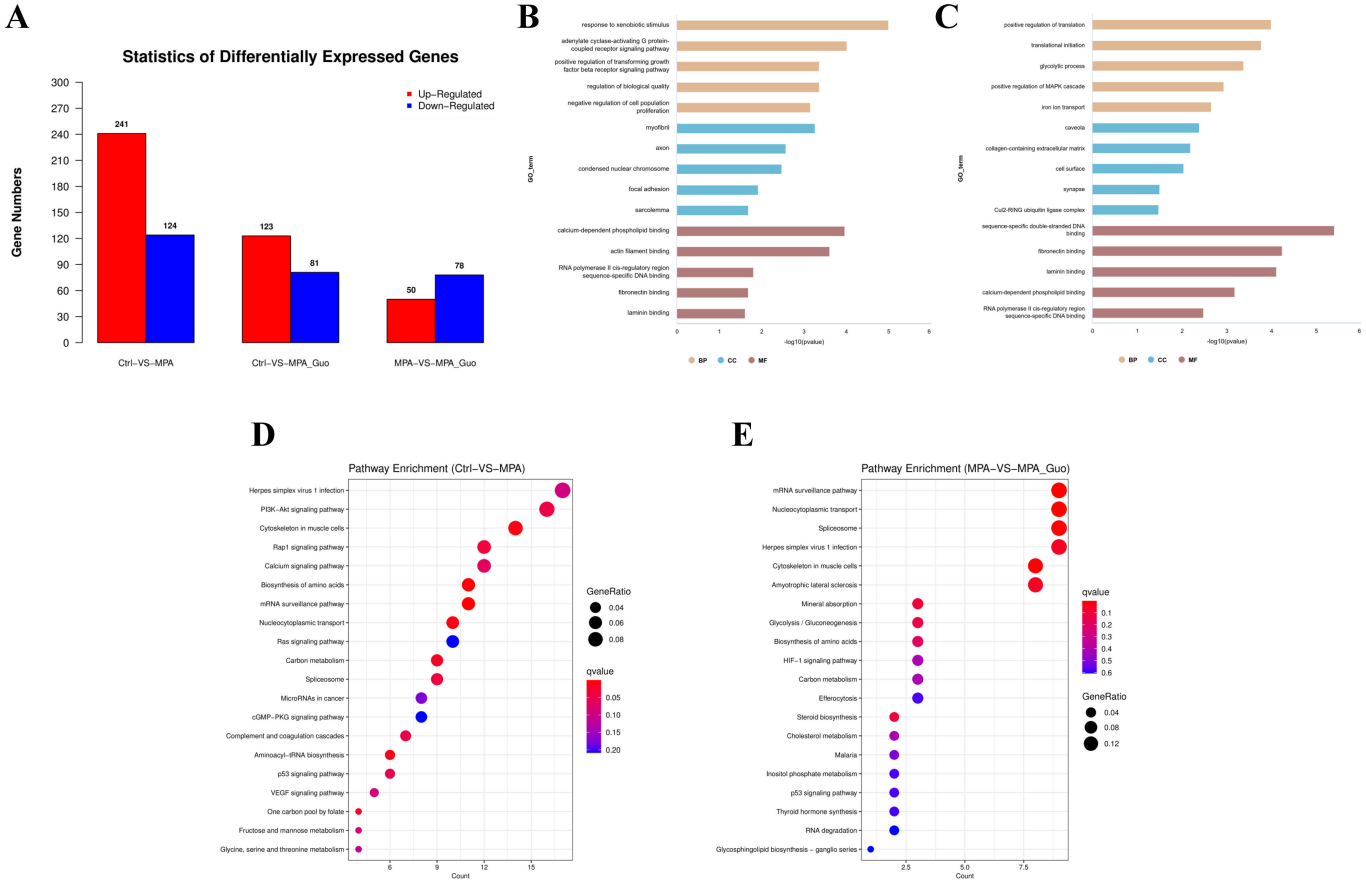
RNA-seq analysis of GUO’s alleviation of MPA induced apoptosis in PGK12.1 ESCs **(A)** Differential gene expression map for Ctrl, MPA, and MPA+GUO. **(B)** GO analysis results for Ctrl-vs-MPA treatment. **(C)** GO analysis results for MPA-vs-MPA+GUO treatment. **(D)** KEGG analysis results for Ctrl-vs-MPA treatment. **(E)** KEGG analysis results for MPA-vs-MPA+GUO treatment.

To explore the damage mechanism of MPA and the protective mechanism of GUO, RNA-seq analysis was performed on PGK12.1 ESCs that were untreated, treated with MPA alone, and cotreated with MPA and GUO. Compared with the untreated group, 241 genes were up regulated and 124 genes were down regulated in the MPA treated group. Compared with the MPA treated group, 50 genes were up regulated and 78 genes were down regulated in the MPA and GUO cotreated group. Compared with the untreated group, 123 genes were up regulated and 81 genes were down regulated in the MPA and GUO cotreated group (**Figure 5A**). The results of GO enrichment analysis showed that pathways such as response to xenobiotic stimulus, positive regulation of transforming growth factor beta receptor signaling pathway, and negative regulation of cell population proliferation were significantly enriched in the Ctrl-VS-MPA group (**Figure 5B**); pathways such as homologous chromosome pairing at meiosis, synaptonemal complex assembly, sulfotransferase activity, and response to toxic substance were significantly enriched in the MPA-VS-MPA+GUO group (**Figure 5C**). The KEGG results indicated that pathways regulating cell proliferation, apoptosis, and differentiation, such as Biosynthesis of amino acids, Ras/Rap1 signaling pathway, PI3K−Akt signaling pathway, and p53 signaling pathway, were significantly enriched in the Ctrl-VS-MPA group; signaling pathways such as mRNA surveillance pathway, spliceosome and p53 signaling pathway were significantly enriched in the MPA-VSMPA+GUO group.

## 4 Discussion

MPA, one of the most commonly used immunosuppressants in organ transplantation and autoimmune diseases, exhibits anti-inflammatory, antiviral, and anticancer activities. Although the effects of MPA on the human body have been widely studied, its impact on developmental processes at the cellular level remains unclear. Embryonic stem cells can be used to model early embryonic development and to investigate the cytotoxic mechanisms of MPA (Allison and Eugui, 2000). Cell proliferation, apoptosis, and differentiation are fundamental biological processes that collectively maintain tissue homeostasis, development, and repair. These processes are regulated by a series of proteins and factors, including cyclins, cyclin dependent kinases (CDKs), caspases, and transcription factors (Basu et al., 2022).

In this study, we morphologically observed that even trace amounts of MPA induced characteristic apoptotic features and significantly inhibited the proliferation of PGK12.1 cells. This finding contrasts with the reduced proliferation observed in HFTF cells treated with MPA at concentrations of 1 mM and 10 mM MMF (Morath et al., 2008), and also differs markedly from the treatment doses required to affect osteosarcoma cells (Wu et al., 2010). This discrepancy may stem from the rapid proliferation of stem cells, which have a higher demand for GTP required for DNA synthesis. As a result, PGK12.1 cells may be more sensitive to MPA induced inhibition of intracellular IMPDH activity than other cell lines. Future studies could investigate the gene expression levels of IMPDH1 and IMPDH2 in PGK12.1 cells to further clarify this mechanism. Meanwhile, since there is no study on the mitigation of MPA induced toxicity by GUO in mouse embryonic stem cells, we designed a preexperiment with a gradient of 0.1–10 mM of GUO with reference to the results of *in vitro* studies in other cell lines and determined 8 mM as the optimal rescuing concentration for the subsequent experiments (Heinz et al., 2003). In previous studies, the optimal action concentration of GUO was mostly centered on 0.1-0.2 mM, while the maximum concentration used in this study was about ~40 times higher (Sagot et al., 2005; Jurkiewicz et al., 2019). This may be due to the lower cellular uptake rate of GUO, necessitating higher concentrations to effectively counteract MPA induced GTP depletion.

The observed down regulation of proliferation-related genes CCND1 and CDK1 suggests that MPA interferes with the normal cell cycle progression of PGK12.1 cells by altering the expression of cell cycle regulatory proteins. Bcl2, an anti-apoptotic protein localized to the outer mitochondrial membrane, endoplasmic reticulum, and nuclear membrane, plays a crucial role in maintaining redox balance via antioxidant pathways. In this study, the in-creased Bax/Bcl2 ratio and elevated caspase-3 levels indicate that MPA compromised the cells’ oxidative stress tolerance. Since all apoptotic signals ultimately converge on caspase-3—the final executioner of apoptosis—its activation leads to a cascade of downstream events, including DNA fragmentation, endonuclease activation, disruption of nuclear and cytoskeletal proteins, protein cross-linking, phagocytic signal expression, and apoptotic body formation (Elmore, 2007). These findings are consistent with our morphological observations and confirm that MPA induces apoptosis in PGK12.1 cells. Moreover, the decrease in mitochondrial membrane potential is considered one of the earliest biological changes during apoptosis. In our experiment, increased ROS levels were detected using the JC-1 assay, suggesting that the molecular mechanism under-lying MPA induced apoptosis may involve elevated oxidative stress via ROS accumulation. This, in turn, may lead to a reduction in mitochondrial membrane potential, caspase-3 activation, DNA damage, and ultimately the inhibition of stem cell proliferation and the induction of apoptosis in mouse embryonic stem cells (Deng et al., 2022).

The differentiation of stem cells into embryonic germ layers represents the initial step in multipotent differentiation. In this study, retinoic acid (RA) served as the principal extrinsic inductive signal driving neural differentiation (Maden, 2007), while Nestin re-mains the canonical marker of neural stem/progenitor cells (NSPCs) (Wiese et al., 2004). Map2 is a marker gene for mature neurons and an ectodermal lineage marker (Dehmelt and Halpain, 2005). Gata6 is the earliest marker of the primitive endoderm, promoting the differentiation of the primitive streak (PS) into mesoderm via BMP and WNT signaling pathways. Eomesodermin (Eomes) interacts with phosphorylated SMAD2/3 to induce endodermal differentiation (Morrisey et al., 1998). PGK12.1 cells underwent a 5-day *in vitro* differentiation protocol, during which the MPA treated group exhibited down regulated expression of Oct4 compared to controls. Oct4 is a key transcription factor maintaining mouse embryonic stem cell (mESC) self-renewal and pluripotency; its down regulation suggests reduced pluripotency. Expression of pluripotency markers typically decreases during stem cell differentiation. Prior studies have shown that ethanol exposure disrupts the balance of Sox2, Oct4, and Nanog in different embryonic stem cell subpopulations, thereby influencing differentiation trajectories (Takahashi and Yamanaka, 2006). Given that mechanisms regulating pluripotency and differentiation may differ, further analysis of differentiation-related gene expression was war-ranted in this study. RT-QPCR results revealed significant up regulation of Nestin, Map2, Gata6, Sox17, and Eomes, indicating successful RA-induced differentiation of PGK12.1 cells. Morphologically, differentiated cells formed rosette-like aggregates consistent with prior reports (Shi et al., 2012; Wang et al., 2016). Based on these observations, we hypothesize that extending RA induction for an additional 3–5 days may yield elongated, fibrous neural cells. MPA treatment resulted in a sequential decrease in germ layer marker gene expression, suggesting inhibition of normal neural differentiation in mouse embryonic stem cells. Within the first two days of MPA exposure, severe cell death was observed in medium- and high-dose groups, with cell counts significantly lower than controls. We speculate that impaired intercellular communication underlies the reduced differentiation capacity of PGK12.1 cells. Additionally, the activation of apoptotic path-ways may further compromise stem cell differentiation (Fujita et al., 2008).

Studies have demonstrated that the proliferation inhibition induced by IMPDH inhibitors can be reversed by supplementing guanosine (GUO), guanosine monophosphate (GMP), guanosine triphosphate (GTP), or deoxy-GMP. Exogenous GUO supplementation is the most commonly employed rescue strategy. For instance, GUO supplementation in human Tenon fibroblasts treated with mycophenolate mofetil (MMF) completely counteracts the drug’s concentration-dependent growth inhibition (Heinz et al., 2003). The MPA induced yeast cell cycle arrest can be reversed by culturing cells in medium supplemented with GUO, which is metabolized to GMP via the Hpt1 enzyme (Liu et al., 2020). Following MPA treatment, decreased tRNA levels were observed in RAW 264.7 macrophages and yeast, with significant recovery upon GMP and GUO supplementation, respectively (Levesque et al., 2006). Therefore, we investigated whether GUO supplementation could alleviate MPA induced cytotoxicity in the PGK12.1 cell line. Exogenous GUO administration at various concentrations reversed MPA induced inhibition of proliferation and differentiation, reduced apoptotic markers, mitigated reactive oxygen species (ROS)-mediated oxidative stress, and restored mitochondrial membrane potential in PGK12.1 cells. These findings suggest that GUO effectively alleviates MPA mediated suppression of growth and differentiation in PGK12.1 cells. At concentrations up to 10 mM, GUO did not further enhance cell viability, possibly reflecting saturation of intracellular GTP utilization.

Our results suggest that MPA may exert teratogenic effects by inducing apoptosis and inhibiting differentiation. However, proliferation, apoptosis, and differentiation are regulated by multiple interconnected signaling pathways rather than a single mechanism. As these mechanisms remain incompletely understood, we employed transcriptome sequencing to further elucidate the relevant pathways. The term “response to xenobiotic stimulus” describes an organism’s defensive or metabolic reactions to exogenous compounds (e.g., drugs, toxins, pollutants), typically involving activation of detoxifying enzymes such as cytochrome P450 or oxidative stress responses (Guengerich, 2008). The transforming growth factor β (TGF-β) receptor pathway mediates down-stream signals (e.g., Smad phosphorylation), regulating differentiation, apoptosis, and fibrosis (Meng et al., 2015). Negative regulation of proliferation involves tumor suppressors (such as p53) and cell cycle inhibitors (such as p21), preventing uncontrolled growth and tumorigenesis (Vousden and Prives, 2009). The PI3K-Akt pathway supports survival, proliferation, and metabolism and is frequently hyperactivated in cancers due to mutations (Manning and Toker, 2017). The p53 path-way responds to DNA damage and promotes apoptosis by activating p21 and pro-apoptotic genes such as Bax, thereby suppressing tumorigenesis (Kastenhuber and Lowe, 2017). GO and KEGG enrichment analyses suggest that MPA toxicity in PGK12.1 cells may involve inhibition of TGF-β receptor and PI3K-Akt pathways, resulting in decreased survival and proliferation, while activating apoptosis and affecting differentiation via the p53 pathway. Following GUO treatment, GO enrichment analysis showed significant enrichment of terms including sulfotransferase activity and response to toxic substances, indicating GUO’s rescuing and reparative effects on MPA induced cellular damage. Enrichment of terms related to homologous chromosome pairing during meiosis and synaptonemal complex assembly suggests that GUO’s rescue mechanisms may involve modulation of chromatin organization and DNA damage response and cytoskeletal dynamics. KEGG pathway analysis further supports this hypothesis, highlighting the pivotal role of the p53 signaling pathway in regulating proliferation, apoptosis, and differentiation. The present study still has some limitations, including high *in vitro* GUO concentration, lack of protein level validation of transcriptome results and *in vivo* experimental evidence. Subsequent studies may further elucidate the mechanism of action and physiological relevance of MPA by thoroughly analyzing its inhibition of IMPDH-induced GTP depletion and its effects on p53 activation and PI3K-Akt pathway suppression.

## 5 Conclusions

This study employed mouse embryonic stem cells as a model to investigate the effects of MPA on preimplantation embryonic development, the mechanisms underlying MPA induced toxicity, and the protective effects of GUO. The results demonstrated that MPA inhibits proliferation, promotes apoptosis, and suppresses differentiation of PGK12.1 cells. GUO alleviates the pro-apoptotic and differentiation-inhibitory effects of MPA on PGK12.1 cells. The cytotoxic effects of MPA on PGK12.1 cells may be mediated by inhibition of signaling pathways such as the TGF-β receptor and PI3K-Akt, leading to reduced cell survival and proliferation, increased apoptosis via the p53 pathway, and impaired differentiation. The protective effect of GUO may operate at the levels of chromatin architecture and cytoskeletal organization. Furthermore, the p53 signaling pathway appears to play a central role in regulating cell proliferation, apoptosis, and differentiation.

## Data Availability

The datasets presented in this study can be found in online repositories. The names of the repository/repositories and accession number(s) can be found in the article/supplementary material.

## References

[B1] Abt E. Rashid K. Le T. Lee H. Creech A. Wu T. . (2022). Abstract 3034: purine nucleoside phosphorylase regulates metabolic and immune checkpoints. Cancer Res. 82, 3034. doi: 10.1158/1538-7445.AM2022-3034

[B2] Allison A. C. Eugui E. M. (2000). Mycophenolate mofetil and its mechanisms of action. Immunopharmacology 47, 85–118. doi: 10.1016/s0162-3109(00)00188-0, PMID: 10878285

[B3] Anderka M. T. Lin A. E. Abuelo D. N. Mitchell A. A. Rasmussen S. A. (2009). Reviewing the evidence for mycophenolate mofetil as a new teratogen: case report and review of the literature. Am. J. Med. Genet. A 149A, 1241–1248. doi: 10.1002/ajmg.a.32685, PMID: 19441125

[B4] Basu S. Greenwood J. Jones A. W. Nurse P. (2022). Core control principles of the eukaryotic cell cycle. Nature 607, 381–386. doi: 10.1038/s41586-022-04798-8, PMID: 35676478 PMC9279155

[B5] Bentley R. (2000). Mycophenolic acid: a one hundred year odyssey from antibiotic to immunosuppressant. Chem. Rev. 100, 3801–3826. doi: 10.1021/cr990097b, PMID: 11749328

[B6] Braun-Sand S. B. Peetz M. (2010). Inosine monophosphate dehydrogenase as a target for antiviral, anticancer, antimicrobial and immunosuppressive therapeutics. Future Med. Chem. 2, 81–92. doi: 10.4155/fmc.09.147, PMID: 21426047

[B7] Cicinnati V. R. Hou J. Lindemann M. Horn P. A. Sotiropoulos G. C. Paul A. . (2009). Mycophenolic acid impedes the antigen presenting and lymph node homing capacities of human blood myeloid dendritic cells. Transplantation 88, 504–513. doi: 10.1097/TP.0b013e3181b0e608, PMID: 19696633

[B8] Dehmelt L. Halpain S. (2005). The map2/tau family of microtubule-associated proteins. Genome Biol. 6, 204. doi: 10.1186/gb-2004-6-1-204, PMID: 15642108 PMC549057

[B9] Deng Y. Zhang Z. Yang H. Wang J. Feng L. Su Y. . (2022). Mycophenolic acid induces the intestinal epithelial barrier damage through mitochondrial ros. Oxid. Med. Cell. Longev. 2022, 4195699. doi: 10.1155/2022/4195699, PMID: 35847589 PMC9277164

[B10] Eckardt K. Stahlmann R. (2010). Use of two validated *in vitro* tests to assess the embryotoxic potential of mycophenolic acid. Arch. Toxicol. 84, 37–43. doi: 10.1007/s00204-009-0476-1, PMID: 19856175

[B11] Elmore S. (2007). Apoptosis: a review of programmed cell death. Toxicol. Pathol. 35, 495–516. doi: 10.1080/01926230701320337, PMID: 17562483 PMC2117903

[B12] Evans M. J. Kaufman M. H. (1981). Establishment in culture of pluripotential cells from mouse embryos. Nature 292, 154–156. doi: 10.1038/292154a0, PMID: 7242681

[B13] Fotie J. (2018). Inosine 5’-monophosphate dehydrogenase (impdh) as a potential target for the development of a new generation of antiprotozoan agents. Mini-Rev. Med. Chem. 18, 656–671. doi: 10.2174/1389557516666160620065558, PMID: 27334467

[B14] Fujita J. Crane A. M. Souza M. K. Dejosez M. Kyba M. Flavell R. A. . (2008). Caspase activity mediates the differentiation of embryonic stem cells. Cell Stem Cell. 2, 595–601. doi: 10.1016/j.stem.2008.04.001, PMID: 18522852 PMC2494585

[B15] Girman P. Lipár K. Kočík M. Voska L. Kožnarová R. Marada T. . (2020). Sirolimus vs mycophenolate mofetil (mmf) in primary combined pancreas and kidney transplantation. Results of a long-term prospective randomized study. Am. J. Transplant. 20, 779–787. doi: 10.1111/ajt.15622, PMID: 31561278

[B16] Guengerich F. P. (2008). Cytochrome p450 and chemical toxicology. Chem. Res. Toxicol. 21, 70–83. doi: 10.1021/tx700079z, PMID: 18052394

[B17] Heinz C. Heise K. Hudde T. Steuhl K. P. (2003). Mycophenolate mofetil inhibits human tenon fibroblast proliferation by guanosine depletion. Br. J. Ophthalmol. 87, 1397–1398. doi: 10.1136/bjo.87.11.1397, PMID: 14609842 PMC1771913

[B18] Jurkiewicz A. Leśniewska E. Cieśla M. Gorjão N. Kantidakis T. White R. J. . (2019). Inhibition of trna gene transcription by the immunosuppressant mycophenolic acid. Mol. Cell. Biol. 40:e00294–19. doi: 10.1128/MCB.00294-19, PMID: 31658995 PMC6908259

[B19] Kastenhuber E. R. Lowe S. W. (2017). Putting p53 in context. Cell 170, 1062–1078. doi: 10.1016/0092-8674(90)90662-x, PMID: 28886379 PMC5743327

[B20] Levesque E. Delage R. Biancamano M. B. Couture F. Guillemette C. (2006). Ugt1a8 and ugt1a9 as molecular determinants of mycophenolate mofetil (mmf) pharmacokinetics. Blood 108, 3191. doi: 10.1182/blood.V108.11.3191.3191

[B21] Liu P. Sarnoski E. A. Olmez T. T. Young T. Z. Acar M. (2020). Characterization of the impact of gmp/gdp synthesis inhibition on replicative lifespan extension in yeast. Curr. Genet. 66, 813–822. doi: 10.1007/s00294-020-01068w, PMID: 32232569 PMC7367712

[B22] Maden M. (2007). Retinoic acid in the development, regeneration and maintenance of the nervous system. Nat. Rev. Neurosci. 8, 755–765. doi: 10.1038/nrn2212, PMID: 17882253

[B23] Manning B. D. Toker A. (2017). Akt/pkb signaling: navigating the network. Cell 169, 381–405. doi: 10.1016/j.cell.2017.04.001, PMID: 28431241 PMC5546324

[B24] Meng X. M. Tang P. M. Li J. Lan H. Y. (2015). Tgf-β/smad signaling in renal fibrosis. Front. Physiol. 6. doi: 10.3389/fphys.2015.00082, PMID: 25852569 PMC4365692

[B25] Morath C. Reuter H. Simon V. Krautkramer E. Muranyi W. Schwenger V. . (2008). Effects of mycophenolic acid on human fibroblast proliferation, migration and adhesion *in vitro* and in *vivo*. Am. J. Transplant. 8, 17861797. doi: 10.1111/j.1600-6143.2008.02322.x, PMID: 18786225

[B26] Morrisey E. E. Tang Z. Sigrist K. Lu M. M. Jiang F. Ip H. S. . (1998). Gata6 regulates hnf4 and is required for differentiation of visceral endoderm in the mouse embryo. Genes. Dev. 12, 3579–3590. doi: 10.1101/gad.12.22.3579, PMID: 9832509 PMC317242

[B27] Pisoni C. N. D’Cruz D. P. (2008). The safety of mycophenolate mofetil in pregnancy. Expert Opin. Drug Saf. 7, 219222. doi: 10.1517/14740338.7.3.219, PMID: 18462179

[B28] Rezvanfar M. A. Hodjat M. Abdollahi M. (2016). Growing knowledge of using embryonic stem cells as a novel tool in developmental risk assessment of environmental toxicants. Life Sci. 158, 137–160. doi: 10.1016/j.lfs.2016.05.027, PMID: 27208651

[B29] Sagot I. Schaeffer J. Daignan-Fornier B. (2005). Guanylic nucleotide starvation affects saccharomyces cerevisiae mother-daughter separation and may be a signal for entry into quiescence. BMC Cell Biol. 6, 24. doi: 10.1186/14712121-6-24, PMID: 15869715 PMC1274246

[B30] Shi Y. Kirwan P. Livesey F. J. (2012). Directed differentiation of human pluripotent stem cells to cerebral cortex neurons and neural networks. Nat. Protoc. 7, 1836–1846. doi: 10.1038/nprot.2012.116, PMID: 22976355

[B31] Spielmann H. Pohl I. Döring B. Liebsch M. Moldenhauer F. (1997). The embryonic stem cell test (est), an *in vitro* embryotoxicity test using two permanent mouse cell lines: 3t3 fibroblasts and embryonic stem cells. Vitro Toxicol. 10, 119–127. doi: 10.1007/978-3-7091-7500-2_69

[B32] Takahashi K. Yamanaka S. (2006). Induction of pluripotent stem cells from mouse embryonic and adult fibroblast cultures by defined factors. Cell 126, 663–676. doi: 10.1016/j.cell.2006.07.024, PMID: 16904174

[B33] Vousden K. H. Prives C. (2009). Blinded by the light: the growing complexity of p53. Cell 137, 413–431. doi: 10.1016/j.cell.2009.04.037, PMID: 19410540

[B34] Wang L. Liu H. Yin Z. Li Y. Lu C. Wang Q. . (2022). A novel guanine elicitor stimulates immunity in arabidopsis and rice by ethylene and jasmonic acid signaling pathways. Front. Plant Sci. 13. doi: 10.3389/fpls.2022.841228, PMID: 35251109 PMC8893958

[B35] Wang Y. Sun Z. Chen S. Jiao Y. Bai C. (2016). Ros-mediated activation of jnk/p38 contributes partially to the proapoptotic effect of ajoene on cells of lung adenocarcinoma. Tumour Biol. 37, 3727–3738. doi: 10.1007/s13277015-4181-9, PMID: 26468015

[B36] Wiese C. Rolletschek A. Kania G. Blyszczuk P. Tarasov K. V. Tarasova Y. . (2004). Nestin expression–a property of multi-lineage progenitor cells? Cell. Mol. Life Sci. 61, 2510–2522. doi: 10.1007/s00018-004-41446, PMID: 15526158 PMC11924557

[B37] Wu J. Izpisua B. J. (2016). Stem cells: a renaissance in human biology research. Cell 165, 1572–1585. doi: 10.1016/j.cell.2016.05.043, PMID: 27315475

[B38] Wu T. Y. Peng Y. Pelleymounter L. L. Moon I. Eckloff B. W. Wieben E. D. . (2010). Pharmacogenetics of the mycophenolic acid targets inosine monophosphate dehydrogenases impdh1 and impdh2: gene sequence variation and functional genomics. Br. J. Pharmacol. 161, 1584–1598. doi: 10.1111/j.1476-5381.2010.00987.x, PMID: 20718729 PMC3010569

[B39] Zhang S. Yang R. Yin N. Zhao M. Li S. Liang X. . (2025). Developmental toxicity and skin sensitization potential of synthetic phenolic antioxidants and butylated hydroxytoluene transformation products: insights from human embryonic stem cell models. J. Hazard. Mater. 492, 138300. doi: 10.1016/j.jhazmat.2025.138300, PMID: 40250273

