# Correction: Guanine nucleoside alleviates mycophenolic acid-induced toxicity in mouse embryonic stem cells

**DOI:** 10.3389/fcimb.2025.1760684

**Published:** 2026-01-05

**Authors:** Baoshan Lin, Ying Ta, Dandan Ou, Xiaolong Liu, Xiaoqiang He, Zhun Rang, Dongqiang Zhang, Wei Fu, Daoliang Lan

**Affiliations:** 1 College of Animal Husbandry and Veterinary Medicine, Southwest University for Nationalities, Chengdu, China; 2 Animal Disease Prevention and Control Center of Aba Tibetan and Qiang Autonomous Prefecture, Markang, China; 3 Science Technology and Agriculture and Animal Husbandry Bureau of Hongyuan County, Hongyuan, China; 4 Longri Stud Farm, Hongyuan, China

**Keywords:** MPA, mouse embryonic stem cells, GUO, proliferation, apoptosis, differentiation, rescue

There was a mistake in **Figure 1D** as published. When we conducted the Western blots to test the protein level of PCNA, Bax, Bcl2, Caspase3 in cells under different concentration of MPA treatment, the same housekeeping protein (tubulin) was used. However, when we exhibited the representative images in **Figure 1D** and **Figure 2F**, the identical picture of tubulin was presented. To avoid the unwanted misunderstandings, we replace the representative images in **Figure 1D** as follows. Of note, the image replacement DO NOT alter the protein gray values showing in **Figure 1F**. The corrected **Figure 1D** appears below.

**Figure 1:**
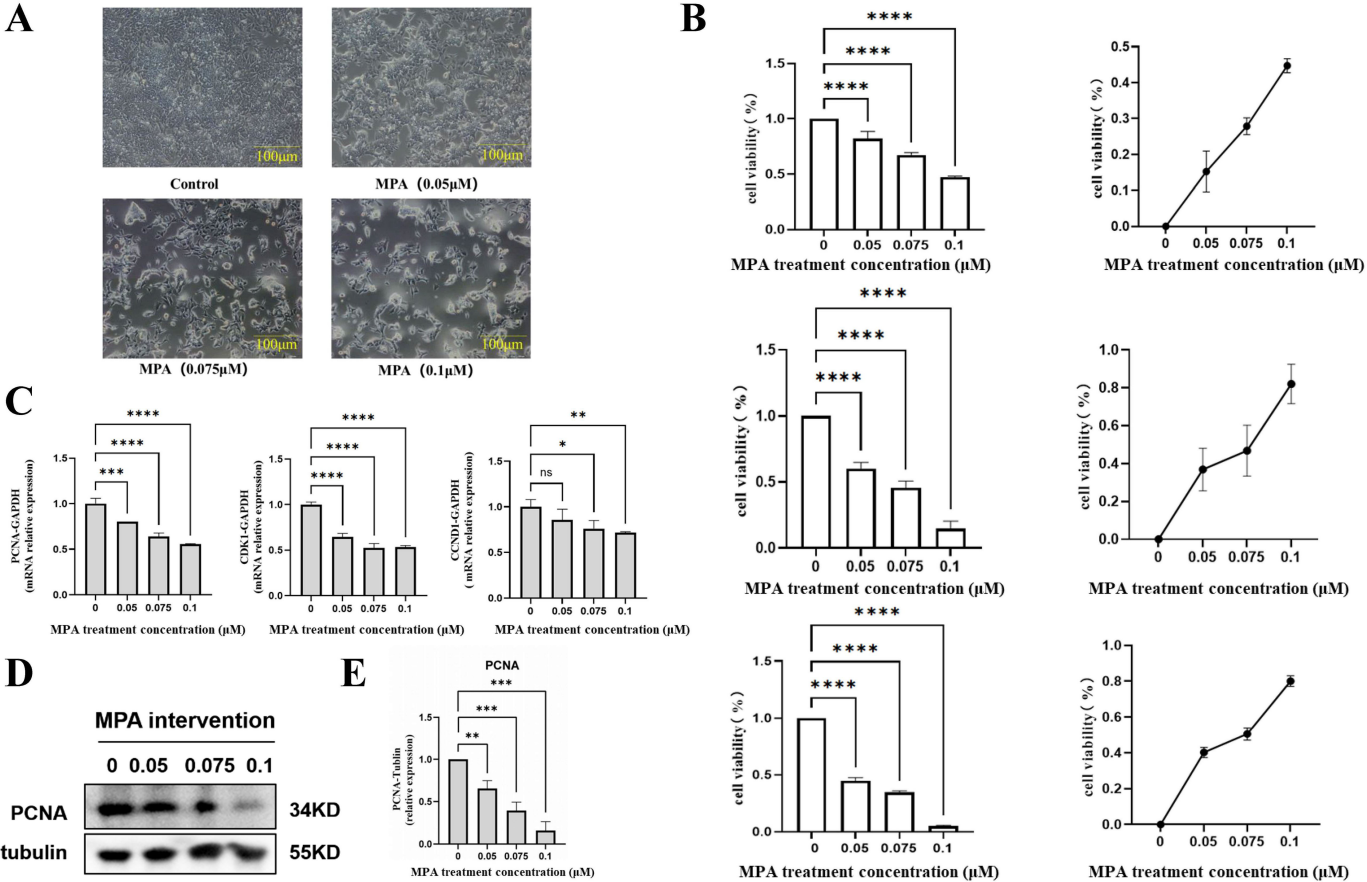
Effect of MPA on the proliferation of PGK12.1 cell line. **(A)** Morphological changes of PGK12.1 cell line after 24 h of MPA treatment (scale bar, 100μm); **(B)** Effects of different concentrations of MPA on cell viability and proliferation inhibition rate of PGK12.1 cells after 24 h, 48 h, and 72 h of treatment. **(C)** Detection of mRNA expression levels of cell proliferation related genes PCNA, CDK1, and CCND1 by RT-QPCR. **(D)** Detection of cell proliferation related protein PCNA expression by Western Blot; **(E)** Quantitative analysis of grayscale values of protein bands. * Indicates a statistically significant difference compared to the corresponding control group, with * representing P < 0.05, ** representing P < 0.01, *** representing P < 0.001, **** representing P < 0.0001, and ns indicating no difference and no statistical significance.

There was a mistake in **Figure 4.1C** as published. When we typeset the images, parts of pictures showing in **Figure 2A** were unconsciously repeated presented in **Figure 4.1C**. Therefore, we replaced the improper images in **Figure 4.1C** as follows. Of note, the right images were used to access the fluorescence intensity of ROS, so the image replacement DO NOT alter the statistical result showing in **Figure 4.1D**. The corrected **Figure 4.1C** appears below.

**Figure 4.1:**
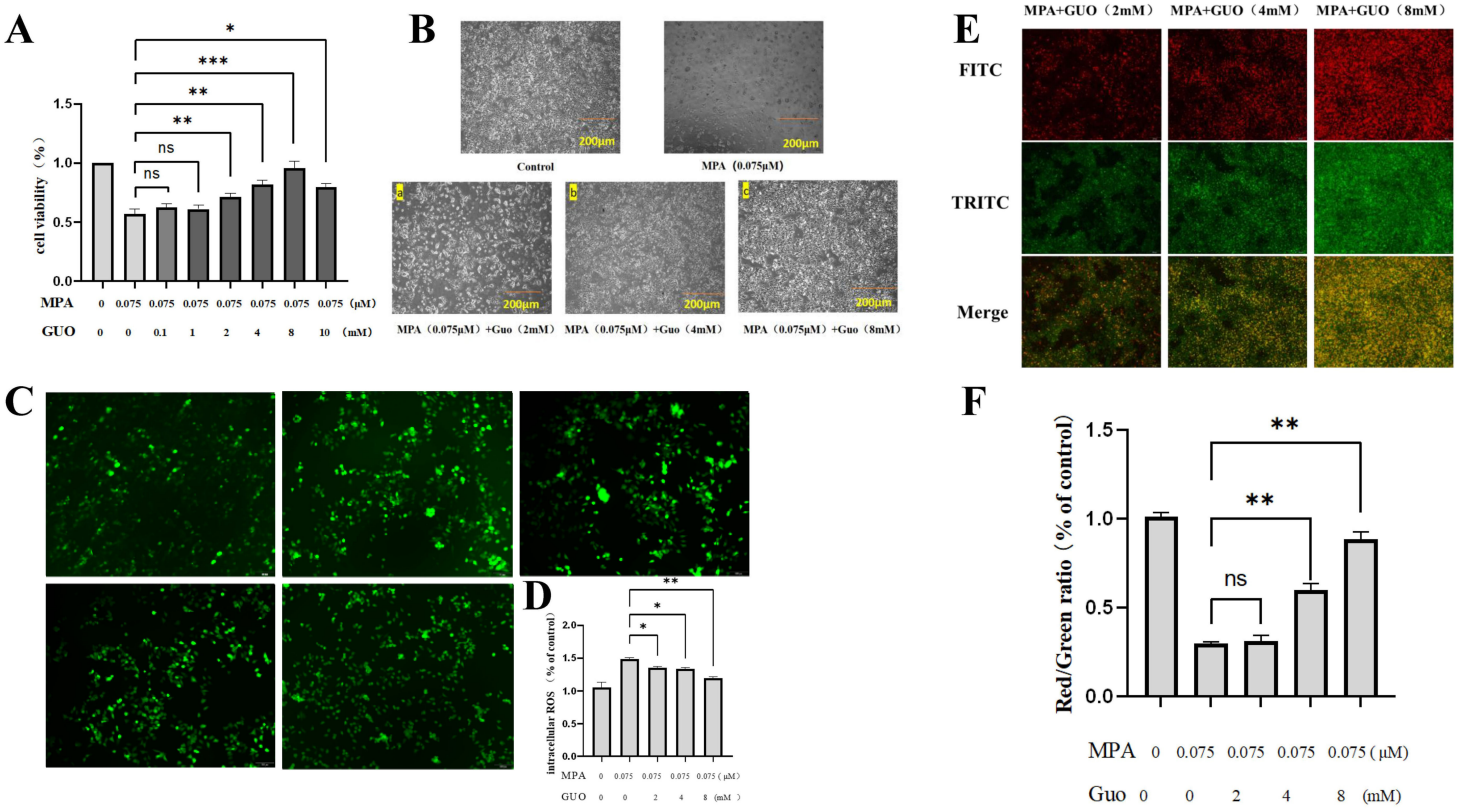
The rescue effect of GUO on MPA treated PGK12.1 cell line. **(A)** Cell viability detection after treating PGK12.1 with different concentrations of GUO + MPA (0.075 uM) for 24 hours. **(B)** The effect of GUO on MPA induced morphological changes in PGK12.1 ESCs (scale bar, 200μm). **(C)** Fluorescent staining results of intracellular ROS content (scale bar, 100μm). **(D)** Analysis of ROS fluorescence intensity. **(E)** JC-1 detection of mitochondrial membrane potential fluorescent staining results in PGK12.1 ESCs (scale bar, 100μm). **(F)** Analysis of the red/green fluorescence intensity ratio of mitochondrial membrane potential. * representing P < 0.05, ** representing P < 0.01, *** representing P < 0.001.

There was a mistake in **Table 1** as published. When we typeset the table contents, parts of the RT-qPCR primers were missing. Therefore, we supplement the information in the following **Table 1**. The corrected **Table 1** appears below. 

**Table 1:** 

Genes	Sequence (5’-3’)
*PCNA*	F: TTGCACGTATATGCCGAGACC; R: GGTGAACAGGCTCATTCATCTCT
*CCND1*	F: ACCCTGACACCAATCTCCT; R: CTCCTTCTGCACGCACTT
*CDK1*	F: GAACACCTTTCCCAAGTGGA; R: CCATTTTGCCAGAGATTCGT
*Bax*	F: TGAAGACAGGGGCCTTTTTG; R: AATTCGCCGGAGACACTCG
*Bcl2*	F: GTCGCTACCGTCGTGACTTC; R: CAGACATGCACCTACCCAGC
*Caspase3*	F: ACAGCACCTGGTTACTATTC; R: CAGTTCTTTCGTGAGCAT
*Gapdh*	F: TGCCCCCATGTTTGTGATG; R: TGTGGTCATGAGCCCTTCC
*Nestin*	F: GGCATCCCTGAATTACCCAA; R: AGCTCATGGGCATCTGTCAA
*Gata6*	F: CTTCTCCTTCTACACAAGCGACCA; R: ATACTTGAGGTCACTGTTCTCGGG
*Map2*	F: TTCTTTTGCTTGCTCGGGATT; R: ATACAGGGCTTGGTTTATTTCAGAGA
*Eomes*	F: CCCTATGGCTCAAATTCCAC; R: CCAGAACCACTTCCACGAAA
*Sox17*	F: ACTTGCTCCCCACAATCACT; R: ACCCCGCTGTTTGTGTTTAG
*Oct4*	F: CCCCAATGCCGTGAAGTTG; R: TCAGCAGCTTGGCAAACTGTT

The original version of this article has been updated.

